# Preparing for the next emerging perinatal infectious disease

**DOI:** 10.1038/s41390-023-02987-3

**Published:** 2024-01-22

**Authors:** Pierre Buekens, Jackeline Alger, Mabel Berrueta

**Affiliations:** 1 Department of Epidemiology, School of Public Health and Tropical Medicine, Tulane University, New Orleans, LA USA; 2 Departamento de Laboratorio Clínico, Hospital Escuela, Tegucigalpa, Honduras; 3 Instituto de Enfermedades Infecciosas y Parasitología Antonio Vidal, Tegucigalpa, Honduras; 4 Instituto de Efectividad Clínica y Sanitaria (IECS), Buenos Aires, Argentina

This special issue of the journal highlights emerging and re-emerging pediatric viral diseases.^1,2^ With climate change, mosquito-borne diseases are spreading, from Zika virus (ZIKV) to dengue virus (DENV) and chikungunya virus (CHKV) infections. A global One Health approach is crucial, linking domestic and international efforts and developing solutions through collaborative actions.^3^ Many emerging infectious diseases are perinatal, affecting pregnant people, infants, and children around the world. A global action plan is needed with strong synergies between disciplines and a perinatal approach linking interventions during pregnancy and childhood.

Hoffman and Maldonado emphasize the need to be ready for “Disease X”.^1^ The World Health Organization (WHO) proposed the concept of “Disease X” to reinforce the preparedness for the emergence of new diseases. We need to be ready to quickly activate perinatal data collection systems to study the impact of emerging viruses on pregnancy and childhood. During the ZIKV epidemic, many cohorts were launched too late and started recruiting after the peak of the epidemic.^4^ Ongoing perinatal data collection systems provide a unique opportunity to collect data quickly if they already have ethical approval and an effective data management system.^5^ To be maintained, such systems should be limited to minimal data sets, with the capability to be modified to add new data quickly. Blood sample collection and storage could also be kept to a minimum and rapidly increased as needed. Protocol modifications should be as straightforward as possible to be submitted to Institutional Review Boards (IRBs) and ethics committees for fast review. The key point is to have all the mechanisms in place before the next viral epidemic strikes. Our cohort on Zika in Pregnancy in Honduras (ZIPH) was initiated in 2016 and continues collecting a limited set of data and one blood sample at the first prenatal visit, in addition to birth outcomes.^6^ Stored blood samples include dried blood spots that can be stored at room temperature, providing a backup in case of power outages. An inventory of scanned barcodes of stored samples is maintained by our data center in Buenos Aires, Argentina, which also prepares enrollment reports on a regular basis. The data center activities are ready to be ramped up in case of an epidemic, including the emergence of a new virus. A data center located in the Global South facilitates south to south collaborations and the use of data collection methods feasible in the southern hemisphere. It is important to have the systems in place and running at a minimal and sustainable level in “peacetime”, so it can be ready when needed.

The ZIKV epidemic highlighted the challenges of accurate measurement of exposure. Serological testing is often complicated by cross-reactivity within the same family of viruses, such as flaviviruses. ZIKV and DENV are transmitted by the same mosquitoes, occur in the same regions, and are difficult to differentiate from symptoms only. The plaque reduction neutralization test (PRNT) is the gold standard for serology but is difficult to perform routinely and is sometimes difficult to interpret.^7^ Another challenge is the availability of laboratories and reagents to perform nucleic acid amplification tests. We used the Centers for Disease Control and Prevention (CDC) Trioplex polymerase chain reaction (PCR) to differentiate ZIKV, DENV, and CHIKV, but its availability remains limited.^8^ The time window to enroll subjects with a positive PCR is often narrow, and having enough power in individual studies is a challenge. Individual participants data (IPD) meta-analyses are a useful approach to pool data from several studies. An example is the IPD on ZIKV infection coordinated by WHO.^4^

Assessment of birth and neurodevelopmental outcomes has several challenges. Measurement errors are an issue for important birth outcomes, such as head circumference. We previously showed a strong digit preference in measuring head circumference at birth, decreasing its usefulness for routine surveillance.^9^ The importance of neurodevelopmental testing is illustrated by several articles in this special issue of the Journal. The Bayley Scales of Infant and Toddler Development, Third Edition (BSID-III) is a gold standard but requires training and standardization; its paper format and copyright restrictions are also a challenge. There is a need for further validation of other simplified instruments which could be easily deployed in the field.

New vaccines for pregnant people and children are available or being developed. Challenges include the lack of time to develop vaccines during an epidemic and the difficulty of testing efficacy after a viral outbreak ends. Additional challenges of some new vaccines include the risk of antibody-dependent enhancement, a major issue for the DENV vaccine, which is only recommended with laboratory-confirmed previous infection.^1^ The Coalition for Epidemic Preparedness Innovations (CEPI) plays a key role in the development of new vaccines. A first live-attenuated CHKV vaccine is now available, and others suitable for future use in pregnancy are in development. Including pregnant people in clinical trials of new vaccines should be a priority but is rarely achieved. We thus often must rely on observational studies to evaluate vaccine effectiveness and safety. Active surveillance systems are being reinforced but do not always include a comparison group of unvaccinated people. Perinatal information systems, including vaccinated and unvaccinated pregnant people, can provide comparative data.

Systematic reviews and meta-analyses need to be made available without delay. Living systematic reviews are continually updated, incorporating relevant new primary evidence as it becomes available. Our living systematic review on COVID-19 vaccines and pregnancy will soon be expanded to CHIKV vaccines in pregnancy and childhood, starting with reviewing vaccine platforms and components.^10^ The web-based interface yields automated proportion meta-analyses with corresponding background rate estimates. It also includes meta-analyses of associations between vaccines and outcomes of interest for studies adjusting for potential confounders. Observational studies on vaccines and pregnancy need to take into account the gestational age at vaccination. Unadjusted association will show a biased protective effect of vaccination on preterm births because of a decreased likelihood of being vaccinated when the gestation is shorter. Not adjusting for socio-economic status might also give the impression that vaccines are protective, assuming that higher socio-economic status is associated with both vaccination and better outcomes. The opposite effect might be observed if vaccination is more frequent in the presence of co-morbidities. Appropriate analyses will decrease the risk of such biases.

It is crucial to maintain the capability of collecting and analyzing data between epidemics. When a new virus emerges, there is no time to develop new study protocols and procedures. Mosquito-borne diseases often spread very quickly in non-immune populations but are often at their peak for a few months only. Speed is of the essence. We need to learn from previous emerging viral infectious diseases to be ready for the ones to come.
